# Pulmonary actinomycosis and pulmonary nocardiosis mimicking lung cancer: A case series and narrative review

**DOI:** 10.17305/bb.2026.14101

**Published:** 2026-04-20

**Authors:** Konstantinos Papatheodosiou, Andreas M Matthaiou, Nikoleta Bizymi, Maria Gangadi, Vasileios Panagoulias, Ioannis Tomos, Serafeim Chrysikos, Adamantia Liapikou

**Affiliations:** 1 5th Department of Respiratory Medicine, Sotiria Thoracic Diseases General Hospital of Athens, Athens, Greece; 2 Laboratory of Molecular and Cellular Pneumonology, Medical School, University of Crete, Heraklion, Greece; 3 Respiratory Physiology Laboratory, Medical School, University of Cyprus, Nicosia, Cyprus; 4 Department of Nursing, School of Health Sciences, National and Kapodistrian University of Athens, Athens, Greece; 5 2nd Department of Respiratory Medicine, Sotiria Thoracic Diseases General Hospital of Athens, Athens, Greece

**Keywords:** *Actinomyces, Nocardia*, pulmonary actinomycosis, pulmonary nocardiosis, rare respiratory infections, lung cancer

## Abstract

Pulmonary actinomycosis and pulmonary nocardiosis are rare chronic respiratory infections that may closely resemble lung cancer due to their overlapping clinical, radiological, and bronchoscopic characteristics. This hybrid case series and narrative review aims to elucidate the presenting patterns, diagnostic pathways, management strategies, and practical implications of these infections in the differential diagnosis of lung cancer. We analyzed four consecutive cases managed at the Sotiria Thoracic Diseases General Hospital in Athens from 2021 to 2025, supplemented by eight additional cases identified through a review of Greek grey literature from the same period. In the 12 cases reviewed, patients commonly presented with cough, dyspnea, fever, hemoptysis, malaise, and weight loss. Chest imaging frequently revealed consolidation, cavitation, atelectasis, nodules, or mediastinal lymphadenopathy, often prompting an oncological evaluation. Bronchoscopic findings occasionally mimicked malignant submucosal spread, and increased fluorodeoxyglucose uptake added to the diagnostic uncertainty. Notably, one case exhibited coexistence of pulmonary actinomycosis with lung adenocarcinoma. Microbiological and histopathological evaluations confirmed the diagnoses, and most patients showed clinical and radiological improvement following prolonged targeted antimicrobial therapy. Given these findings, pulmonary actinomycosis and nocardiosis should be considered in patients with suspected lung cancer, particularly when infectious features coexist with mass-like or cavitary lesions. Careful tissue sampling, specialized microbiological testing, and structured follow-up are essential for excluding occult or coexisting malignancy.

## Introduction

Pulmonary actinomycosis and nocardiosis are rare chronic respiratory infections caused by *Actinomyces spp.* and *Nocardia spp.*, respectively [1, 2]. Both conditions closely resemble lung cancer, which can result in delayed diagnosis and treatment, presenting challenges in clinical practice [3–10]. *Actinomyces* is a Gram-positive, non-acid-fast, anaerobic, branching filamentous bacterium [1, 11]. The incidence of pulmonary actinomycosis has declined due to widespread antibiotic use, with an estimated annual incidence of approximately 1 per 3,000,000 individuals, representing a minority of actinomycosis cases [11]. Risk factors for this infection include alcohol abuse, poor oral hygiene, and chronic aspiration [1, 11, 12]. In contrast, *Nocardia* is a Gram-positive, partially acid-fast, aerobic, branching filamentous bacterium that can act as both an opportunistic and primary pathogen [2, 13]. The estimated annual incidence of nocardiosis ranges around 1 per 2,500,000 individuals [13]. Nocardiosis is strongly associated with immunosuppression, such as that seen in prolonged corticosteroid treatment, tissue or organ transplantation, and solid or hematological malignancies; however, it can also occur in immunocompetent patients [2, 13, 14]. Both infections predominantly affect middle-aged to older adults, with a mean age of 50–65 years [11, 13].

These conditions often present with non-specific clinical findings, including productive cough, hemoptysis, chest pain, dyspnea, fever, night sweats, malaise, anorexia, and unintentional weight loss—symptoms that frequently overlap with those of lung malignancies and other chronic respiratory infections [1, 2]. Additionally, radiological findings are often non-specific; chest computed tomography (CT) may reveal consolidations with or without cavitation, ground-glass opacities, nodules or masses, atelectasis, and hilar or mediastinal lymphadenopathy, as well as possible pleural thickening and pleural effusion [1, 3, 15]. Due to these non-specific clinical and radiological findings, both infections may prompt extensive oncological work-ups, including positron emission tomography (PET)–CT and invasive sampling, which can lead to diagnostic and therapeutic dilemmas [5, 9]. Furthermore, distinguishing pulmonary actinomycosis and nocardiosis from malignancy is complicated by overlapping bronchoscopic features [16, 17]. Pulmonary actinomycosis may manifest as mass-like lesions or endobronchial abnormalities that mimic malignant disease, while nocardiosis can produce cavitating nodules or masses and metabolically active lesions on PET imaging [6, 7, 9, 10, 15, 16]. Importantly, literature documents instances of coexistence between either infection and malignancy [16–18]. A structured follow-up is essential to identify potential coexistence or occult malignancy. Consequently, tissue sampling, thorough microbiological work-up—including cultures and, where feasible, molecular diagnostics—and systematic radiological follow-up are critical to avoid missed cancer diagnoses and to ensure complete resolution of suspicious lesions [1, 2, 11, 13].

In this context, we present a series of four cases of pulmonary actinomycosis and nocardiosis managed at the Sotiria Thoracic Diseases General Hospital of Athens, the largest referral center for respiratory diseases in Greece. This series highlights the diagnostic pitfalls that arise when these infections masquerade as lung cancer, including cases of intense fluorodeoxyglucose (18F-FDG) avidity and mediastinal lymphadenopathy, as well as documented coexistence with lung adenocarcinoma [5, 9, 18, 19]. Additionally, we compiled and synthesized cases from hospitals across the country reported in grey literature sources over the past five years, aiming to describe presenting patterns, diagnostic pathways, management approaches, and practical implications for cancer exclusion and follow-up.

## Methods

This study comprises a hybrid case series from the 5th Department of Respiratory Medicine at the Sotiria Thoracic Diseases General Hospital of Athens and a grey literature review of unpublished cases in Greece.

All consecutive cases of pulmonary actinomycosis and nocardiosis identified by attending physicians over a five-year period (2021–2025) were recorded. Resemblance to lung cancer was determined based on any of the following three criteria: i) clinical presentation, including chronicity of symptoms and the presence of constitutional symptoms such as anorexia, unintentional weight loss, or malaise; ii) chest imaging findings, including the presence of one or more space-occupying lesions with or without cavitation; and iii) bronchoscopic findings, including lesions resembling malignant submucosal spread or exophytic endoluminal lesions.

The grey literature in Greece was searched for case reports of pulmonary actinomycosis and nocardiosis diagnosed and treated across the country over the last five years (2021–2025). The search included the Abstract Books (in Greek or English) of the Panhellenic Pulmonology Congress, which is the largest scientific event in Respiratory Medicine in Greece organized annually by the Hellenic Thoracic Society, and the National Archive of Ph.D. Theses in Greece (in Greek or English). The search strategy utilized the terms “*Actinomyces*,” “actinomycosis,” “*Nocardia*,” and “nocardiosis” across all fields. All records describing one or more cases of pulmonary infection by *Actinomyces spp.* or *Nocardia spp.* were included as eligible. Extracted relevant data included the isolated pathogen, demographics, smoking history, alcohol consumption, oral hygiene of the patients, immunocompetency, clinical manifestations, diagnostic tests, radiological and bronchoscopic findings, treatment (regimen and duration), and outcomes.

It is important to emphasize that, although this work was not a systematic review, the aim of searching the grey literature was to identify additional cases not previously reported in the international literature, thereby enriching this case series to more clearly delineate the clinical relevance of pulmonary actinomycosis and nocardiosis in relation to lung cancer. To the best of our knowledge, all identified case reports were confirmed to be unpublished elsewhere.

### Ethical statement

This study adhered to the principles outlined in the Declaration of Helsinki, and written informed consent has been obtained from the patients to publish this paper.

## Case series

### Case 1

**Figure 1.**
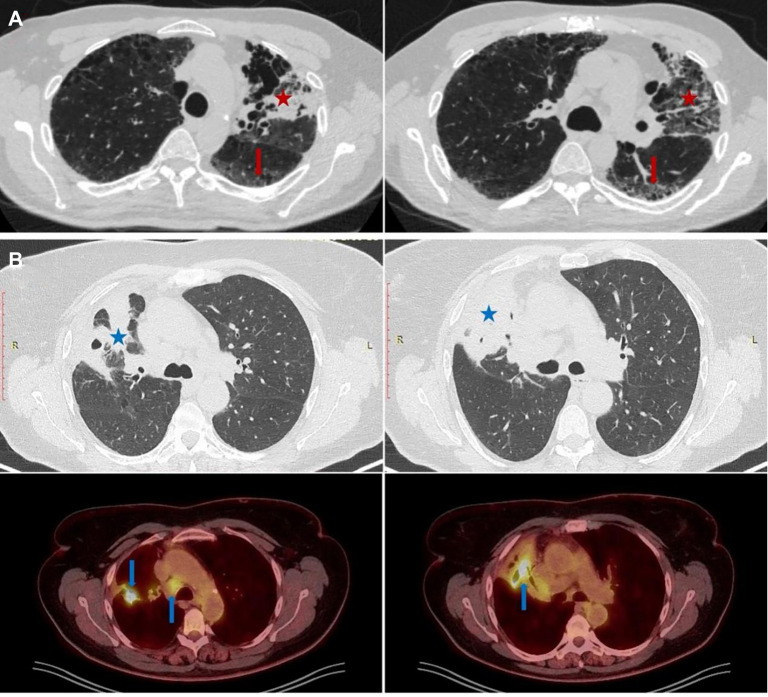
**Typical radiological findings from chest CT and PET–CT in cases of pulmonary actinomycosis, as demonstrated in Cases 1 and 2.** (A) Case 1. Chest CT revealed a large consolidation with extensive cavitation in the left upper lung lobe (red asterisks), accompanied by usual interstitial pneumonia characterized by subpleural honeycombing and traction bronchiectasis, predominantly observed in the lower lung lobes (red arrows). **(**B) Case 2. Chest CT (upper row) displayed extensive consolidation and atelectasis in the right upper and middle lung lobes, along with cavitation and mediastinal lymphadenopathy (blue asterisks). PET–CT (lower row) indicated significant increased 18F-FDG uptake (blue arrows) in the consolidative lesion of the right upper lung lobe (SUV_max_ ═ 10.7) and mediastinal lymph nodes (SUV_max_ ═ 4.1 in the right lower paratracheal, 3.5 in the subcarinal, and 2.9 in the right hilar nodal stations). Abbreviations: ^18^F-FDG, fluorine-18 fluorodeoxyglucose; CT, computed tomography; PET–CT, positron emission tomography–computed tomography; SUVmax, maximum standardised uptake value.

A 64-year-old male, an ex-smoker with a 40 pack-year history and unremarkable past medical history, presented with fever, exertional dyspnea, and a non-productive cough lasting two weeks. Symptoms did not improve despite the administration of empirical antimicrobial therapy. Chest CT at admission revealed a large consolidation with extensive cavitation in the left upper lung lobe, accompanied by typical interstitial pneumonia, including subpleural honeycombing and traction bronchiectasis predominantly in the lower lung lobes (Figure 1A). Systemic inflammatory markers were elevated, while serum immunoglobulins, autoantibodies, and tumor markers yielded unremarkable results. Flexible bronchoscopy demonstrated patency of the tracheobronchial tree to the level of the subsegmental bronchi, with no endoluminal lesions, and abundant purulent secretions were noted, primarily in the left upper lung lobe, where bronchoalveolar lavage (BAL) was performed. A Gram-positive branching bacterium isolated from the BAL fluid culture was identified as *Actinomyces israelii*, while mycobacterial infection was excluded. A dentist assessed oral hygiene, revealing no dental lesions. Given the profound clinical presentation and the extensive consolidative lesion depicted on chest imaging, colonization by the pathogen was deemed unlikely. The patient was discharged on a prolonged course of high-dose oral amoxicillin (1 g TID) for 12 months, and antifibrotic therapy was also initiated. Follow-up visits and imaging indicated both clinical improvement and radiological regression of the cavitary lesion.

**Figure 2.**
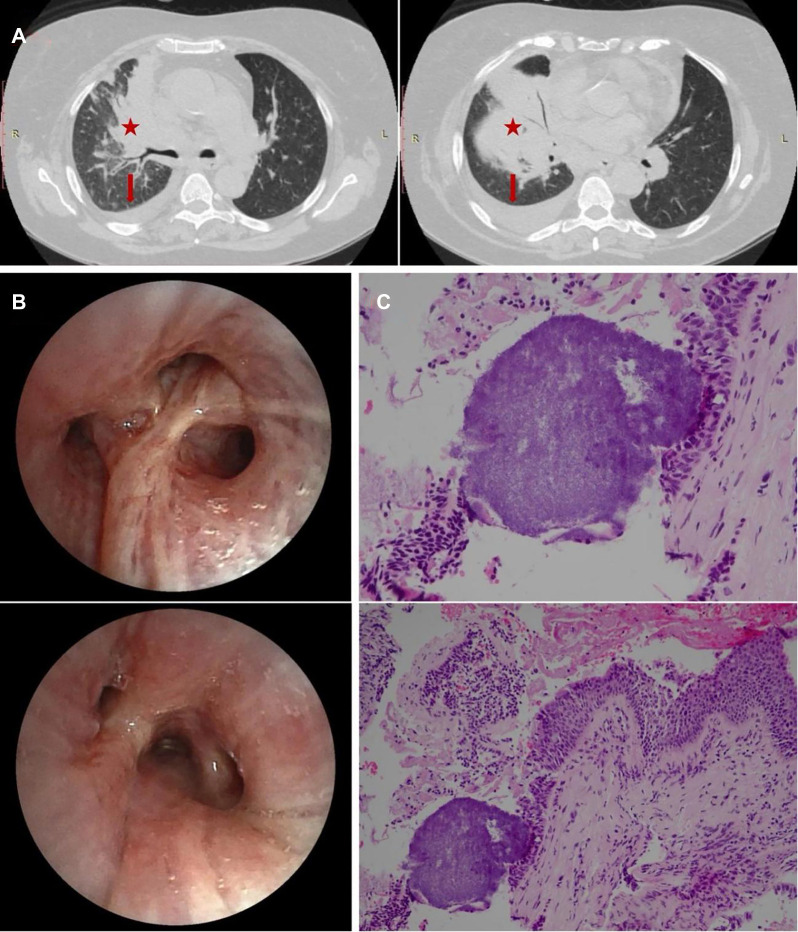
**Radiological (chest CT), bronchoscopic, and histopathological (transbronchial biopsy, TBB) findings of pulmonary actinomycosis coexisting with lung adenocarcinoma, as illustrated in Case 3.** (A) Radiological findings. Chest CT exhibited dense atelectasis with air bronchograms in the RUL (red asterisks) and an ipsilateral pleural effusion (red arrows). (B) Bronchoscopic findings. Flexible bronchoscopy revealed edematous orifices of the right upper and middle lobar bronchi, displaying concentric narrowing indicative of malignant submucosal spread. Microbiological testing of bronchial washings for common pathogens, mycobacteria, and fungi yielded negative results. (C) Histopathological findings. Histopathological examination of TBB from the middle lobe demonstrated lung adenocarcinoma (cytokeratin 7-positive, p40-negative, and thyroid transcription factor 1-negative) alongside filamentous bacterial colonies consistent with *Actinomyces spp*. Neoplastic cells varied from small to medium in size and exhibited hyperchromatic nuclei, eosinophilic cytoplasm, and intracellular mucin. Mild inflammatory cell infiltration was noted. Abbreviations: CT, computed tomography; RUL, right upper lobe; spp., species; TBB, transbronchial biopsy.

### Case 2

A 54-year-old female, a current smoker with a history of 60 pack-years, was admitted for the evaluation of a subacute productive cough characterized by purulent sputum, recurrent hemoptysis, and unintentional weight loss of approximately 10 kg over the past two months. The patient also reported recurrent lower respiratory tract infections that did not respond to oral empirical antimicrobial therapy with a macrolide. Her past medical history was unremarkable, and neither immunosuppression nor poor oral hygiene were identified. A chest CT scan revealed extensive consolidation and atelectasis in the right upper lung lobe (RUL) and middle lung lobe, cavitation in the RUL, and mediastinal lymphadenopathy (Figure 1B). With a working diagnosis of lung malignancy based on clinical manifestations and radiological findings, a CT-guided fine needle biopsy (FNB) of the lesion was performed. Histopathological results indicated fibrinopurulent exudate without evidence of malignancy.

A subsequent PET–CT scan demonstrated increased 18F-FDG uptake in the consolidative lesion of the RUL (maximal standardized uptake value, SUVmax = 10.7) and mediastinal lymph nodes (SUVmax = 4.1 in the right lower paratracheal, 3.5 in subcarinal, and 2.9 in right hilar nodal stations) (Figure 1B). Flexible bronchoscopy revealed patency of the tracheobronchial tree up to the subsegmental bronchi, with no endoluminal lesions but abundant purulent secretions predominantly in the RUL. Transbronchial biopsies (TBB) from the RUL and endobronchial ultrasound (EBUS)-guided transbronchial needle aspiration (TBNA) from the right lower paratracheal and subcarinal nodal stations were obtained. Histopathological examination of the TBB sample demonstrated multiple sulfur granules and colonies of *Actinomyces spp.*, consistent with pulmonary actinomycosis, while microbiological testing of bronchial washings for common pathogens, mycobacteria, and fungi yielded unremarkable results.

Antimicrobial therapy with piperacillin/tazobactam was initially initiated due to the patient’s severe clinical condition. The regimen was later switched to a combination of meropenem and linezolid due to persistent fever and clinical deterioration. After treatment escalation, defervescence occurred, and the patient was discharged on high-dose oral amoxicillin (1 g TID) for a total of 12 months. Clinical and radiological improvements were gradually observed in follow-up chest imaging.

### Case 3

A 67-year-old female, a current smoker with a history of 60 pack-years and an unremarkable past medical history, was transferred from a regional hospital for further evaluation of progressively worsening dyspnea and non-productive cough. Broad-spectrum antibiotics (piperacillin/tazobactam and doxycycline) were administered without adequate clinical response. The patient was afebrile and hypoxemic upon admission. Laboratory tests revealed elevated systemic inflammatory markers, while blood, sputum, and urine cultures were unremarkable. Chest CT showed dense atelectasis with air bronchograms in the RUL and an ipsilateral pleural effusion (Figure 2A). Flexible bronchoscopy revealed edematous orifices of the right upper and middle lobar bronchi with concentric narrowing, consistent with malignant submucosal spread (Figure 2B). Microbiological testing of bronchial washings for common pathogens, mycobacteria, and fungi was negative. Histopathological examination of TBB from the middle lobe confirmed lung adenocarcinoma (cytokeratin 7-positive, p40-negative, and thyroid transcription factor 1-negative) with coexistent filamentous bacterial colonies consistent with *Actinomyces spp.* (Figure 2C). Antimicrobial therapy was escalated to meropenem and linezolid due to new-onset fever. Administration of chemotherapy was contraindicated due to the patient’s poor performance status. The patient ultimately succumbed in the following weeks due to adenocarcinoma progression, large malignant pleural effusion, and pulmonary embolism. While no definitive histopathological findings of active infection, such as profound inflammatory infiltration and tissue invasion, were observed, the extensive bronchial mucosal inflammation and the response to sole antimicrobial therapy without antineoplastic treatment suggested a possible coexistence of lung malignancy and actinomycosis rather than mere colonization by *Actinomyces spp.*

### Case 4

An 86-year-old female, a never smoker with a history of uterine malignancy during her 50 s and type 2 diabetes mellitus, was admitted for investigation of a two-month history of intermittent low-grade fever, productive cough with purulent sputum, malaise, and unintentional weight loss. The patient had been hospitalized twice in the previous two months and had received broad-spectrum antibiotics. Recent chest CT revealed extensive bilateral alveolar infiltrates with a nodular appearance. The patient was afebrile and hypoxemic upon admission. Laboratory tests indicated elevated systemic inflammatory markers and normocytic normochromic anemia. No pathogens were isolated in blood, sputum, or urine cultures.

A follow-up chest CT interestingly revealed multiple patchy alveolar opacities with a migratory pattern compared to the previous imaging. Flexible bronchoscopy and BAL were performed, revealing predominantly lymphocytic cellular populations (20.6%) in the BAL fluid. In the interim, while awaiting microbiological results, oral prednisolone was initiated at a dose of 0.5 mg/kg/day, based on a working diagnosis of organizing pneumonia derived from clinical, radiological, and cytological findings. Testing for serum immunoglobulins, autoantibodies, and tumor markers yielded unremarkable results.

Notably, while mycobacterial and fungal infections were excluded, BAL culture for common pathogens yielded *Nocardia spp.* A brain CT was performed, ruling out central nervous system involvement. Antimicrobial therapy with trimethoprim/sulfamethoxazole (TMP/SMX) at a dose of 15 mg/kg/day was selected for a 12-month duration, alongside a tapering corticosteroid regimen. A follow-up chest CT performed four weeks after admission demonstrated almost complete resolution of the multiple patchy alveolar opacities.

## Other reported cases

In addition to the previously discussed cases, eight additional cases were identified in the grey literature [20–23]. These included five patients diagnosed with pulmonary infections caused by *Actinomyces spp.* and three by *Nocardia spp.* (Table 1) [24–31].

**Table 1:** Reported cases of pulmonary actinomycosis and nocardiosis, including clinical background, manifestations, diagnostic investigations, and management strategies

**Authors and year of report [Ref.]**	**Pulmonary infection**	**Pathogen**	**Sex**	**Age (*years*)**	**Ever smoking**	**Alcohol abuse**	**Poor oral hygiene**	**Immune compromise**	**Clinical manifestations**
Patsouras & Manos, 2021 [34]	Actinomycosis	*A. odontolyticus*	Male	50	Yes	Yes	Yes	No	Fever, productive cough
Kechris et al., 2023 [35]	Actinomycosis	*Actinomyces spp.*	Female	72	Yes	No	ND	Yes	Fever, dyspnoea, diarrhoea
Assioura et al., 2024 [36]	Nocardiosis	*Nocardia spp.*	Male	82	ND	ND	ND	Yes	Dyspnoea, weight loss
Gkerpini et al., 2024 [37]	Nocardiosis	*Nocardia spp.*	Female	57	ND	ND	ND	No	Low-grade fever, productive cough
Mavrokefalos et al., 2024 [38]	Actinomycosis	*Actinomyces spp.*	Male	65	Yes	ND	ND	No	Fever, dyspnoea
Mermigkis et al., 2024 [39]	Actinomycosis	*A. graevenitzii*	Male	70	ND	ND	ND	Yes	Fever, malaise, respiratory failure
Papavasileiou et al., 2024 [40]	Nocardiosis	*N. cyriacigeorgica*	Female	58	Yes	ND	ND	No	Recurrent fever, dyspnoea
Rampiadou et al., 2024 [41]	Actinomycosis	*Actinomyces spp.*	Male	76	Yes	ND	Yes	No	Dyspnoea
**[Ref.]**	**Resemblance of lung cancer**	**Imaging tests**	**Radiological findings**	**Interventional investigation**	**Bronchoscopical findings**	**Initial empirical therapy**	**Final targeted Therapy**	**Duration (*months*)**	**Outcome**
[34]	No	CT	RLL cavitated consolidation	Bronchoscopy	Endoluminal suppurative lesions	PIP/TAZ Linezolid	AMP/SAM Doxycycline	9	Improvement
[35]	Yes	CT	RUL mass and mediastinal lymphadenopathy	Bronchoscopy EBB CT-guided FNB	Endoluminal exophytic lesion with lumen obstruction	Multiple regimens	Ampicillin	1–2	Complete resolution
[36]	No	CT	Bilateral cavitated nodules and GGO	None	None	Meropenem Voriconazole	TMP/SMX	ND	ND
[37]	Yes	CT	LLL consolidation	Bronchoscopy EBB	Endoluminal exophytic lesion with lumen obstruction	Multiple regimens	TMP/SMX	6	Complete resolution
[38]	Yes	CT	RLL mass	Bronchoscopy EBUS EBUS–TBNA	Right hilar and mediastinal lymphadenopathy	AMOX/CLAV Cefditoren Moxifloxacin	Amoxicillin	12	Improvement
[39]	Yes	PET–CT	Bilateral hypermetabolic pulmonary nodules with central cavitation	Bronchoscopy BAL	Chronic infection	Multiple regimens	AMOX/CLAV Linezolid	12	Complete resolution
[40]	No	CT	RML bronchiectasis, consolidations, and nodules; Lingula & RLL consolidations	Bronchoscopy	Suppurative secretions	β-lactam Macrolide	TMP/SMX Doxycycline	2	ND
[41]	Yes	CT	Right loculated pleural effusion, right lung atelectasis	Bronchoscopy EBB	Endoluminal exophytic lesion with lumen obstruction	Ciprofloxacin Clindamycin	Ciprofloxacin Clindamycin	3	Improvement

Abbreviations: A., *Actinomyces*; AMOX/CLAV, amoxicillin/clavulanate; AMP/SAM, ampicillin/sulbactam; BAL, bronchoalveolar lavage; CT, computed tomography; EBB, endobronchial biopsy; EBUS, endobronchial ultrasound; EBUS–TBNA, endobronchial ultrasound-guided transbronchial needle aspiration; FNB, fine-needle biopsy; GGO, ground-glass opacity; LLL, left lower lobe; N., *Nocardia*; ND, not described; PET–CT, positron emission tomography–computed tomography; PIP/TAZ, piperacillin/tazobactam; Ref., reference; RLL, right lower lobe; RML, right middle lobe; RUL, right upper lobe; spp., species; TMP/SMX, trimethoprim/sulfamethoxazole.

The median age of the five patients with pulmonary actinomycosis was 70 years (range 50–76), with 80% being male and reporting a positive smoking history. Notably, two of these patients were immunocompromised due to type 2 diabetes mellitus or long-term corticosteroid and azathioprine therapy for myasthenia gravis. Two patients had poor oral hygiene, and one also presented with alcohol overuse. Fever and dyspnea were the most common clinical manifestations. Chest CT was the preferred imaging modality, with PET conducted in combination with CT in one case. All patients underwent bronchoscopy and histopathological assessment, revealing a range of findings including suppurative lesions, endoluminal exophytic lesions causing lumen obstruction, and hilar and mediastinal lymphadenopathy. Among the reported species, *A. graevenitzii* and *A. odontolyticus* were isolated from cultures of respiratory secretions. Importantly, complete resolution or substantial improvement of the lesions was observed in all cases following the completion of long-term, targeted antimicrobial therapy.

Conversely, the median age of patients with pulmonary nocardiosis was 58 years (range 57–82), with 67% being female and one patient reporting a positive smoking history. Pulmonary nodules, some with cavitation, were the predominant radiological findings on chest CT scans. A 58-year-old female presenting with recurrent fever and dyspnea underwent bronchoscopy, resulting in the identification of *N. cyriacigeorgica*, while the other two patients reported both respiratory and constitutional symptoms, with *Nocardia spp.* isolated from respiratory secretions. One of these patients was immunocompromised due to hematological comorbidities. All patients demonstrated improvement following treatment with TMP/SMX and/or doxycycline. Notably, none of the patients exhibited any extrathoracic manifestations, including central nervous system involvement.

## Pulmonary actinomycosis and nocardiosis: Similarities and differences

Among rare respiratory infections, pulmonary actinomycosis and nocardiosis present significant diagnostic challenges due to their potential clinical, radiological, and bronchoscopic similarities to lung cancer. A summary of the similarities and differences between these two infections is provided in Table 2.

**Table 2:** A comparative analysis of clinical similarities and differences between pulmonary actinomycosis and nocardiosis

**Microbial or clinical feature**	**Pulmonary actinomycosis** **(*Actinomyces spp.*)**	**Pulmonary nocardiosis** **(*Nocardia spp.*)**
**Microbial phenotype**		
**Metabolism**	Anaerobic	Aerobic
**Gram stain**	Gram-(+) bacilli with branching filamentous hyphae (indifferentiable)	
**Acid-fast culture**	Not acid-fast	Weakly acid-fast
**Sulphur granules**	More common	Less common
**Reservoir and associated exposures**	Oropharyngeal, gastrointestinal, and genitourinary flora	Soil, water, and decaying organic matter
**Pulmonary infection**		
**Clinical background**	Commonly history of orofacial abscess, dental trauma or surgery, or aspiration risk factors	Commonly history of primary or secondary immunosuppression
**Typical clinical manifestations**	Subacute respiratory and constitutional signs and symptoms (indifferentiable): chest pain, dyspnoea, productive cough, and haemoptysis, along with fever, rigors, night sweats, malaise, and unintentional weight loss Additional findings: chest wall masses, pleural effusion (empyema), sinus tracts or pleurocutaneous fistulas draining pus from the chest wall	Subacute respiratory and constitutional signs and symptoms (indifferentiable): chest pain, dyspnoea, productive cough, and haemoptysis, along with fever, rigors, night sweats, malaise, and unintentional weight loss Additional findings: neurological signs and symptoms in immunocompromised patients; cutaneous nodular lesions in immunocompetent patients
**Extrapulmonary involvement**	Commonly local invasion of tissues; haematogenous dissemination less common	Commonly haematogenous dissemination, especially to central nervous system (brain abscess) with or without skin involvement
**Radiological investigation (typical chest computed tomography findings)**	Mass-like consolidation, with frequent cavitation, commonly mimicking tuberculosis or malignancy; tendency to cross tissue planes; pleural and/or chest wall spread; fistulisation	Multiple nodules, with frequent cavitation, commonly mimicking tuberculosis or malignancy; abscess formation; “tree-in-bud” pattern
**Microbiological investigation**	Diagnostic yield improves with culture of BALF or tissue biopsy, beyond sputum, and alertness of the lab	
**Interventional investigation**	Lung biopsy via bronchoscopy or imaging-guided FNB may aid diagnosis	
**Antimicrobial therapy**	Drug of choice: oral penicillin or amoxicillin Alternatives: other beta lactams, doxycycline, and clindamycin Prolonged duration of treatment (weeks–months or more than a year in selected cases)	Drug of choice: oral TMP/SMX Alternatives: ceftriaxone, imipenem, amikacin, and linezolid Prolonged duration of treatment (weeks–months or more than a year in selected cases)
**Interventional management**	Abscess or empyema drainage and fistula or sinus tract excision may be required	Abscess drainage may be required
**Multidisciplinary team approach**	Variably includes: pulmonologist, interventional pulmonologist, infectious disease specialist, thoracic surgeon, radiologist, interventional radiologist, microbiologist, and pathologist	
**Follow-up**	Always necessary, by means of serial chest imaging, to exclude possibly coexistent occult malignancy	
**Common pitfalls**	Empirical management with a misdiagnosis of pneumonia from common pathogens using short, inefficient antimicrobial regimens Delayed diagnosis due to time-consuming investigation with a working misdiagnosis of lung malignancy Insufficient follow-up during and after completion of antimicrobial therapy missing possible occult malignancy	

Abbreviations: BALF, bronchoalveolar lavage fluid; FNB, fine-needle biopsy: Spp., species; TMP/SMX, trimethoprim/sulfamethoxazole.

### Pulmonary actinomycosis

*Actinomyces* is a Gram-positive, fast-acid, anaerobic, branching filamentous bacterium. More than 30 species have been identified, with *Actinomyces israelii* and *Actinomyces genecseriae* being the most common causes of pulmonary infections. Infections can occur in multiple anatomical sites beyond the lungs, including the maxillofacial, cervical, abdominal, and pelvic regions. These bacteria are part of the normal flora of the oropharynx and the gastrointestinal and urogenital tracts, and infection typically follows aspiration, tissue damage, disruption of mucosal barriers, or local invasion of adjacent tissues [1, 11]. Factors such as alcoholism, poor oral hygiene, and an increased risk of aspiration are associated with higher rates of pulmonary actinomycosis [1, 11, 12]. Although primarily reported in adults, pulmonary actinomycosis has also been documented in the pediatric population [32]. Opportunistic pulmonary actinomycosis has been observed in atypical clinical scenarios, including pulmonary alveolar proteinosis and, rarely, congenital lung malformations like pulmonary sequestration [33, 34].

Pulmonary actinomycosis is characterized by non-specific clinical findings, complicating initial diagnosis. Symptoms typically include productive cough, hemoptysis, chest pain, dyspnea, fever, malaise, and night sweats, which can mimic lung malignancies or other chronic respiratory infections, making exclusion essential for appropriate management. An additional diagnostic hurdle is the lack of specific radiological findings. Chest CT may reveal variable consolidations, with or without cavitation, ground-glass opacities, atelectasis, and hilar or mediastinal lymphadenopathy. Less common findings include pleural thickening and effusion, which can present as empyema. Bronchoscopic examination may reveal irregular granular thickening that resembles malignant submucosal spread or exophytic endoluminal masses with purulent exudate. A specialized brush for anaerobic conditions is necessary in cases of high suspicion. Direct endobronchial biopsy or TBB can be performed, particularly under EBUS guidance when available. For mass-like lesions or those adjacent to the chest wall, surgical or CT-guided FNB may be indicated [3–8, 16, 18, 35, 36].

Diagnosis is confirmed through microbiological, histopathological, or molecular detection of the pathogen. Specimens obtained via soft tissue biopsies or deep-needle aspiration can facilitate microbiological diagnosis. These specimens must be rapidly transported to the laboratory under strict anaerobic conditions within 15 min of acquisition. While Gram staining can aid in the direct confirmation of pulmonary actinomycosis, pathogen isolation may require extended culture periods of up to three weeks. The hallmark histopathological finding of actinomycosis is chronic granulomatous infection characterized by sulfur granules, which are yellowish formations measuring 0.1–1 mm in diameter. These represent microcolonies with a basophilic center surrounded by an eosinophilic perimeter, known as the Splendore–Hoeppli phenomenon. While sulfur granules can also be present in other infections, such as nocardiosis, they are highly indicative of actinomycosis [1, 11]. Among molecular assays, polymerase chain reaction (PCR) is the cornerstone of culture-independent diagnosis. Additional diagnostic options include metagenomic next-generation sequencing (mNGS) and matrix-assisted laser desorption/ionization time-of-flight (MALDI–TOF) mass spectrometry. A bronchoalveolar lavage fluid (BALF) sample is commonly preferred for molecular diagnostics [1, 35].

Management of pulmonary actinomycosis primarily involves prolonged administration of β-lactam antibiotics. Mild-to-moderate cases are treated with oral penicillin V (2–4 g per day) or amoxicillin (1.5–3 g per day). In cases of suspected polymicrobial infection, amoxicillin/clavulanate (2 g per day) is preferred. Treatment duration typically lasts 2–6 months, with close monitoring for clinical and radiological improvement. In severe or invasive cases (e.g., extensive necrosis/abscess, pleural involvement/empyema, fistulization, or massive hemoptysis), initial high-dose intravenous therapy with benzylpenicillin, ceftriaxone, or, in cases of suspected polymicrobial infection, piperacillin/tazobactam is administered for 2–4 weeks before transitioning to oral therapy for a total duration of 6–12 months [1, 11, 37].

### Pulmonary nocardiosis

*Nocardia* is a Gram-positive, partially acid-fast, aerobic, branching filamentous bacterium that can act as both an opportunistic and a primary pathogen. This ubiquitous organism is commonly found in water, soil, dust, and decomposing organic matter [2, 13]. *Nocardia* primarily affects the lungs and is more frequently seen in immunocompromised individuals, including those on glucocorticoids or other immunosuppressive therapies, individuals with HIV, and those with solid or hematological malignancies or organ transplants, although cases in immunocompetent patients are increasing. Additional risk factors include diabetes mellitus, alcoholism, chronic obstructive pulmonary disease, bronchiectasis, and tuberculosis [13, 14, 38].

Multisystem involvement is observed in approximately 32% of cases, with lung involvement seen in 39%, central nervous system involvement in 9%, skin involvement in 8%, and other systems (e.g., eyes and bones) in 12%. Pulmonary nocardiosis may present acutely, subacutely, or chronically and can be accompanied by respiratory symptoms, such as dyspnea, pleuritic chest pain, productive cough, and hemoptysis, as well as constitutional symptoms, including fever, night sweats, anorexia, unintentional weight loss, and malaise [2, 13]. Non-specific radiological findings on chest CT may include variable consolidations, nodules or masses (often with cavitations), ground-glass opacities, bronchiectatic lesions, and pleural effusion [13, 15].

Confirmation of nocardiosis can be achieved through the detection of the pathogen in respiratory samples, including sputum, BALF, or lung tissue. Although direct Gram staining may reveal branching Gram-positive filamentous rods, culture techniques, similar to those for *Actinomyces*, are slow and may yield negative results. Molecular methods, including PCR and mNGS, have significantly enhanced both sensitivity and speed of diagnosis. Nevertheless, distinguishing colonization from confirmed infection remains challenging, particularly in patients with chronic underlying conditions [2, 17, 39].

Antimicrobial therapy for pulmonary nocardiosis typically involves a combination of one to three antibiotics. TMP/SMX serves as the cornerstone of treatment, often in combination with amikacin or imipenem. Monotherapy with TMP/SMX may be appropriate in non-severe cases. A standard intravenous dose of 15 mg/kg/day is initiated, with careful monitoring of renal function and drug levels due to the risk of toxicity. Linezolid is also considered an alternative option. Treatment duration is generally prolonged, ranging from 6–12 months, particularly for immunosuppressed patients and those with central nervous system involvement. Maintenance treatment continues for 2–6 weeks, and after clinical improvement is noted, a switch from intravenous to oral therapy is implemented. Optimal management of coexisting chronic lung diseases, such as chronic obstructive pulmonary disease (COPD) and bronchiectasis, or underlying immunosuppression is highly recommended [2, 40].

## Pulmonary actinomycosis and nocardiosis as lung cancer mimickers

Pulmonary actinomycosis and nocardiosis are recognized in the literature as potential mimickers of lung cancer due to overlapping clinical and radiological findings. The phenomenon of lung cancer mimickers is well-documented; infectious or inflammatory entities can present on chest CT as mass-like consolidations, cavitations, atelectasis, pleural involvement, or lymphadenopathy, closely resembling malignant disease. Patients with either infection may exhibit non-resolving pneumonia, hemoptysis, unintentional weight loss, malaise, and mass-like or cavitary findings, complicating differentiation from lung cancer [3, 4, 10]. The convergence of nonspecific clinical and radiological findings may initially trigger an oncological diagnostic pathway, potentially resulting in delayed diagnosis and treatment of the infection, as well as unnecessary procedures and examinations. Therefore, diagnosis based solely on these nonspecific findings is challenging, and histopathological confirmation may be essential [1, 2, 4, 10]. Our case series illustrates this clinical overlap across multiple instances. In Case 1, a former smoker presented with a large cavitary upper lobe consolidation that was resistant to empirical antibiotics. Notably, Case 2 featured a patient’s clinical presentation that included recurrent hemoptysis and unintentional weight loss, alongside upper-lobe consolidation/atelectasis, cavitation, and mediastinal lymphadenopathy. In the case reported by Mavrokefalos et al., imaging revealed a soft-tissue mass with enlarged mediastinal lymphadenopathy, and EBUS–TBNA samples identified multiple *Actinomyces spp.* colonies [28].

A further source of diagnostic uncertainty is the limited discriminatory value of advanced imaging techniques, particularly 18F-FDG PET–CT. Increased 18F-FDG uptake may not effectively differentiate between lung malignancy and actinomycosis when clinical suspicion for both conditions is high [5, 41]. Case 2 exemplifies this pitfall, as the increased 18F-FDG uptake in a lesion with consolidation of the right upper lobe and mediastinal lymph nodes was ultimately determined not to correlate with lung malignancy. PET–CT should primarily serve as a tool for selecting specific areas for precise tissue sampling rather than for confirming or ruling out malignancy [5]. Nocardiosis has also been documented to masquerade as lung malignancy on PET–CT, underscoring that increased metabolic avidity should not be equated with neoplasia [9].

Definitive exclusion of malignancy often necessitates escalation and, at times, repetition of tissue sampling, especially when initial suspicion of malignancy is high [1, 2]. In Case 2, an initial CT-guided FNB yielded non-diagnostic results, while bronchoscopy with TBB revealed sulfur granules and *Actinomyces spp.* colonies. EBUS–TBNA was employed in Case 2 to investigate mediastinal lymphadenopathy. EBUS bronchoscopy plays a crucial role in the differential diagnosis of mediastinal lymphadenopathy [19]. Interestingly, in Case 4, systemic symptoms combined with nodular/migratory opacities resulted in significant diagnostic ambiguity, necessitating close monitoring. Radiological resolution on follow-up after targeted therapy supported an infectious rather than malignant etiology.

A structured and thorough follow-up plan is essential for the definitive exclusion of occult or coexisting malignancy. Case 3 illustrated the presence of lung adenocarcinoma alongside filamentous colonies of *Actinomyces spp.* The diagnosis of a respiratory infection does not preclude the presence of another coexisting clinical entity, including malignancy, particularly in smokers and in cases of persistent atelectasis, pleural effusion, endobronchial abnormalities, or nodal enlargement [1, 11]. Importantly, pulmonary actinomycosis and nocardiosis may initially be misclassified as lung cancer [10, 41]. Follow-up should be comprehensive, including regular reassessment of clinical and radiological findings. Repeated chest CT scans should demonstrate significant reduction or complete resolution of lesions. In cases of incomplete resolution, persistent mass-like lesions, interval growth, new lesions, recurrent hemoptysis, persistent atelectasis, endobronchial abnormalities, or ongoing symptoms, reevaluation of the possibility of malignancy with repeat bronchoscopy or targeted biopsies, potentially including EBUS–TBNA or CT-guided sampling, should be performed [1, 2].

## Conclusion

Pulmonary actinomycosis and nocardiosis represent two rare respiratory infections characterized by a combination of subacute respiratory and constitutional symptoms, which may extend beyond the lungs and often resemble the radiological and bronchoscopic findings of lung cancer. Given their rarity, the need for specialized microbiological techniques to detect the causative pathogens, the requirement for long-term antimicrobial therapy, and the potential for extrapulmonary involvement and complications, these infections can be underdiagnosed or diagnosed with delay, leading to adverse clinical outcomes. This hybrid case series and narrative review of the literature aims to highlight significant diagnostic pitfalls and enhance clinical awareness rather than to endorse broad diagnostic or management recommendations for pulmonary actinomycosis and nocardiosis. Clinical suspicion and specialized training are imperative for the improved identification, treatment, and monitoring of these conditions.
